# Mothers With Postpartum Psychiatric Disorders: Proposal for an Adapted Method to Assess Maternal Sensitivity in Interaction With the Child

**DOI:** 10.3389/fpsyt.2019.00471

**Published:** 2019-07-22

**Authors:** Christine Heinisch, Mirijam-Griseldis Galeris, Sandra Gabler, Susanne Simen, Juliane Junge-Hoffmeister, Judith Fößel, Gottfried Spangler

**Affiliations:** ^1^Developmental and Educational Psychology, Institute of Psychology, Friedrich-Alexander University Erlangen-Nürnberg, Erlangen, Germany; ^2^Department of Paediatric Psychiatry, Psychotherapy and Psychosomatics, Universitätsklinikum Leipzig, Leipzig, Germany; ^3^Klinik für Psychiatrie und Psychotherapie, Universitätsklinik der Paracelsus Medizinischen Privatuniversität, Klinikum Nürnberg Süd, Nürnberg, Germany; ^4^Klinik für Psychotherapie und Psychosomatik des Universitätsklinikums Carl Gustav Carus der Technischen Universität Dresden, Dresden, Germany

**Keywords:** maternal sensitivity, postpartum depression (PPD), postpartum anxiety, mother–child interaction, AINSWORTH

## Abstract

About 15% of mothers suffer from postpartum psychiatric disorders, such as depression, anxiety, or psychosis. Numerous studies have shown maternal caregiving behavior to be negatively affected under these circumstances. The current study sets out to shed light on specific caregiving behaviors of affected mothers in the context of parental mental illness at an early stage. There are several methods to assess maternal caregiving behavior in terms of sensitivity. However, all of them have limitations regarding the peculiarities of mothers with postpartum disorders, that is, changes in affect regulation, and the early onset of the disorder postpartum. With the current study, we provide an adapted method to assess maternal sensitivity based on methods recently approved in attachment research. Two groups of mothers, who were either healthy or had different postpartum disorders, were recorded on video during interactions with their infants. Behaviors were rated regarding responsiveness, promptness, appropriateness, intrusiveness, and positive and negative affect. A first analysis revealed an increased number of deficits on all subscales in mothers with postpartum psychiatric disorders as compared to healthy mothers. Depressive mothers with a single diagnosis had lower scores in responsiveness, promptness, and appropriateness and higher scores on intrusiveness as compared to those in healthy mothers. Here, maternal behavior appears more parent-centered, whereas affect seemed to be relatively unharmed. Moreover, as compared to healthy mothers, mothers with comorbid depression and anxiety symptomatology achieved lower scores on responsiveness, appropriateness, and positive affect and higher scores on intrusiveness and negative affect. It is suggested, that increased deficits are related to the severity of illness in mothers with comorbidities. Results on promptness indicate that these mothers are still capable of maintaining higher vigilance to infant cues. Variance in maternal behavior was relatively high in clinical mothers, showing that some of them are well capable of behaving in a sensitive manner toward their child. One strength of our adapted method is that particular aspects of sensitive parent–child interactions are assessed separately. This may shed light on specific behavior patterns of different postpartum psychiatric disorders, which may in turn relate to specific child outcomes. The manual is open for usage, while reliability testing is required.

## Theoretical Background

### Postpartum Mood Swings and Psychiatric Disorders

The birth of a child is typically represented as a fascinating and happy event. In reality though, a few days after child birth, about 40–80% of mothers experience symptoms of depression and emotional lability (1–3). This phenomenon, commonly known as “baby blues,” usually declines after a couple of days without psychiatric treatment. Tremendous changes in hormonal levels and psychological difficulties in adapting to the new situation after childbirth are supposed to be responsible for this emotional status (4–7). However, when these symptoms manifest in a mental disorder during pregnancy or postpartum, as it does in 10–15%, treatment is mandatory (8, 9). Maternal risk factors are a history of (familial) psychiatric disorders, sociodemographic variables, such as early motherhood, low income, low educational level, and low social support, as well as experiences of maltreatment during childhood or later life (10–13).

The most common and significant diagnoses of postpartum psychiatric disorders are postpartum depression (14), followed by postpartum anxiety (15, 16), postpartum obsessive–compulsive disorder (OCD) (17), and postpartum psychosis (18). Additional compulsive infant-focused thoughts are common in perinatal mental illness (19). Postpartum diagnoses often appear comorbid [e.g., Refs. (20, 21). In sum, they are comparable to disorders outside the postpartum period that refer to alterations in affect and cognition. However, additional peculiarities are that fears, obsessive thoughts, and sense of guilt focus on the child [e.g., Refs. (22–24)].

### Maternal Sensitivity in Mentally Ill Mothers

Various symptoms with postpartum onset interfere with the demands of caring for a child and handling motherhood. Therefore, mothers with postpartum disorders often exhibit specific behavioral restraints during interaction with their infant. More specifically, postnatally depressed mothers, as compared to healthy mothers, show fewer positive caregiving behaviors, less emotional involvement, and less responsiveness when interacting with their children (25). In contrast, anxious mothers show more exaggerated behaviors and increased arousal when interacting with their children (26). Some mothers suffering from compulsive thoughts in terms of harming their child tend to withdraw from interactions as they are afraid to hurt their child (27, 28). In postpartum psychosis, Hornstein and colleagues (29) describe affected mothers as quickly overstrained, emotionally tense, uncoordinated, and sometimes awkwardly moving their infant.

In sum, postpartum psychiatric disorders compromise a mother’s ability to interact with her child in a sensitive way, which decreases the quality of the mother–child interaction [e.g., Refs. (25, 26, 30)]. Sensitive parenting behavior means to consider the child’s perspective, adequately infer and respond to emotional needs, and provoke positive reciprocity during interaction. Sensitivity itself is defined by the *perception*, *correct interpretation*, and a *prompt* and *adequate* reaction to a child’s signals and communication attempts (31). There is evidence that sensitivity is affected in mothers with postpartum disorders. As prior work suggests, there seem to be symptom-specific patterns regarding alterations on different subscales of sensitivity, which are described below.

*Responsiveness*. The perception and reaction toward the child’s signal was found to be lowered and less contingent in mothers with postpartum depression (25, 32–35). Also, maternal trait anxiety reduces responsiveness in healthy mothers (36, 37), whereas, in contrast, clinically anxious mothers show some overactivity (38).

*Appropriateness* of maternal behavior can be observed if an interaction sequence is “well-rounded” by being supportive and child-centered in an adequate way. Such supportive behavior is reduced in mothers with depressive symptoms (33). Anxious mothers behave less appropriately (39) and are more open to misinterpretations of children’s cries, which may then lead to inappropriate reactions (40). Also, repeating sorrowful thoughts can lead to lower structuring during mother–child interactions (26, 41–43). For postpartum psychosis, results regarding the appropriateness of maternal behavior are ambiguous. A recent review postulates that mothers with postpartum psychosis are as likely as postnatally depressed mothers to harm their children (44), whereas Riordan et al. (45) reported observations of greater interaction deficits than those with affective disorders, namely being more remote, insensitive, intrusive and self-absorbed.

Another important component closely related to the concept of sensitivity is *intrusiveness*. Higher scores on this scale are used when the caregiver denies the child’s autonomy and acts in a self-centered controlling way. On the other hand, a nonintrusive caregiver considers the child’s perspective an important part of its individuality and respects it. Mothers with postpartum depression were found to exhibit behaviors of intrusiveness and withdrawal inconsistently [e.g., Refs. (16, 25)]. Highly anxious mothers show more intrusive behaviors compared to mothers with low anxiety levels [e.g., Refs. (36, 37, 43)] as less support of the infants’ autonomy (46). Similarly, at an older age, teenagers with parents suffering from OCD report to be confronted with more controlling behavior and boundaries from their parents (47). Finally, reactions of psychotic mothers toward their children are assumed to be more intense in their intrusive behavior (48).

Several methods used to assess parental interactional behavior emphasize the importance of warmth and positive climate, although these aspects are not included in the original definition of sensitivity. Key aspects of depression are depressed mood and loss of interest. Thus, mothers with this diagnosis often experience blunted emotionality or an increase in negative affect. These emotional characteristics compromise the affective climate during mother–child interactions. More precisely, depression lowers the mothers’ capability to flexibly switch between affective states and adjust to their child’s altering affective states and needs sensitively (49, 50). Also, increases in hostility and negativity could even be observed in mothers who only exhibit subclinical levels of depression (34, 51). Finally, depressed mothers are described as less warm, more irritable, and lack joy in interacting with their child (35, 52).

Negative affect in mothers with postpartum anxiety disorder reveals itself in stressful and sorrowful behavior (53), increased criticism, and less satisfaction regarding the child’s behavior (54, 55). Some studies also describe mothers with postpartum anxiety disorders as less warm and positive in contact with their children, indicated by fewer smiles or positive gestures (46, 54, 56, 57). However, other studies suggest that maternal anxiety may also yield more positive interactions (26) or that maternal affect is at least comparable to healthy mothers (58–60).

The relevance of maternal sensitivity is evidenced by numerous studies that identify this domain of caregiving behavior as an important predictor of the child’s attachment (61–66). Indeed, the quality of attachment is highly sensitive to the appropriateness of early experiences (67, 68). In this line, insecure attachment strategies can be viewed as adaptions to less emotionally available or reliable caregivers, which means that they are functional in a given environment (69). Secure attachment relationships, however, provide the child with a range of competencies and have a positive impact on its social and language development (66, 70–72). With regard to maternal depression, a lack of sensitivity can yield insecure attachment, whereas maternal sensitive behavior can buffer negative effects of depression and increase the likelihood of establishing a secure attachment relationship (73, 74). Interventions focusing on improving maternal sensitivity have the potential to increase the offspring’s attachment security (75). However, specific associations between certain aspects of sensitivity and child outcome variables are still unknown, which is why more fine-grained analyses on a subscale level are needed. Finally, this may be especially important with regard to behavioral impairments exhibited by mentally ill mothers.

### Previous Measures of Maternal Sensitivity

Observational tools to assess maternal behavior in mother–child interactions are numerous (76–79). Most combine scales measuring sensitivity with other observable behaviors like affect or even play and generate an overall score. In the following, we describe the four most influential methods that a) assess parenting behavior in terms of maternal sensitivity and b) have successfully been used to assess predictors of children’s attachment security.

First, the Ainsworth Maternal Sensitivity Scale is one of four scales assessing parental behavior (31): Sensitivity versus Insensitivity to the baby’s signals, Cooperation versus Interference with baby’s ongoing behavior, Physical and Psychological availability versus Ignoring and Neglecting, and Acceptance versus Rejection of the baby’s needs. Here, the scale sensitivity turned out to be a key variable because it was found to be correlated to acceptance, cooperation, and availability and to be a reliable predictor of attachment security (61). Affect is considered in the Acceptance versus Rejection Scale because it deals with the balance between the mother’s positive and negative feelings about the baby. The Ainsworth Maternal Sensitivity Scale requires the observer to make one global assessment of sensitivity. In further analyses, it has been related to attachment quality and a range of child behavior. It is the origin of other developing measurement tools like the NICHD–Study of Early Child Care Mother–Child Interaction (ECCN) Scale.

Second, the NICHD-ECCN Scale for mothers of children from 6 up to 24 months quantifies different aspects of maternal behavior rated from videotaped dyadic semistructured play interactions. It assesses the following aspects of parenting behavior: *Sensitivity/Responsivity to distress* (adapted from the original Ainsworth Maternal Sensitivity Scale), *Sensitivity/Responsivity to nondistress*, *Intrusiveness, Detachment*, *Stimulation of development, Positive regard for the child, Negative regard for the child*, and *Flatness of affect* (80). Even though there are two separate global rating scales measuring maternal sensitivity/responsivity, most studies use a composite measure of overall maternal sensitivity as an indicator of mothers’ sensitive behavior [e.g., Ref. (81)]. The NICHD-ECCN sensitivity scales are one of eight widely used observational measures; however, there are indeed a total of 50 measures related to the construct of maternal sensitivity (79). A number of studies have shown that maternal sensitivity measured by the NICHD-ECCN Scale is predictive of different child outcomes, for example, attachment security (82, 83) or cognitive competence (84).

Third, Biringen and colleagues (85–87) developed the emotional availability (EA) Scales. The EA Scales describe the caregiver’s ability to perceive and react to both positive and negative emotions, as well as the child’s reaction to the caregiver’s behavior. It includes a multidimensional set of features (e.g., caregiver’s sensitivity, nonhostility, structuring, nonintrusiveness). Moreover, the child’s behavior is observed, too. This leads to the evaluation of the affective quality of parent–child relationships from the first weeks of life to adolescence. There is no given structure for the observational situation, and it has been applied in separation–reunion situations (62), still face procedure (88), and structured and semistructured play situations (89, 90).

Fourth, the CARE-Index is a screening tool intended to enable trained professionals to make judgments about the necessity of a family intervention (91). Similar to the EA Scales, the method evaluates the quality of caregiver–infant interaction. Observational situations are 3 min of play interaction recorded on video under nonthreatening conditions. It is applicable from birth to 15 months and up to 2.5 years when using the toddler form. The coding procedure focuses the observer’s attention on seven aspects of the caregiver’s and the infant’s behaviors, including affect and cognition. For caregivers, these are sensitivity, control, and unresponsiveness. These codes define four patterns of caregivers’ interactional behavior. The “sensitive pattern” involves how the caregiver accommodates to the infant’s behavior and shares the most commonalities to the concept of sensitivity. The “controlling pattern” identifies behaviors that are either overtly hostile or covertly hostile comparable to intrusiveness and hostility in other scales, whereas the “unresponsive pattern” consists of items describing forms of withdrawal.

### Limitations of Existing Methods for Mentally Ill Mothers

With many other methods, but the four named as influential in attachment research, it should be just a matter of choice to evaluate maternal sensitivity in postpartum psychiatric disorders. Indeed, all of them have been in use for clinical research [e.g., Refs. (76, 79] with their strengths and weaknesses. However, to integrate sensitivity research and specific parenting characteristics with postpartum psychiatric disorders, each of the named methods has its own limitations.

First, the onset of postpartum depression starts either already during pregnancy with signs for mental health problems or manifestations of depression and anxiety or immediately after birth or at least very few weeks or months after delivery. In practice, the diagnosis is given with diagnostic criteria until children reach the age of 1, in some hospitals even 2. Here, it is important to note that sensitivity measures vary regarding recommendations on the child’s age range: Whereas the scales of Ainsworth, EA, and CARE-Index allow observation from birth on, the NICHD Scale starts from 6 months of age and was not specifically intentionally developed for newborns or younger infants. Moreover, the CARE-Index and the EA Scales measure dyadic interaction, which increases in older infants. This might account for the number of null findings in studies using the EA Scales in depressed and nondepressed mothers (86).

Second, the development from and application with regard to attachment theory and research are different. The CARE-Index and the EA Scales can be regarded as further developments of the Ainsworth sensitivity scales and are both very promising in evaluating the dyadic interaction within the developing attachment relationship because sensitive maternal behavior is related to secure attachment. According to Bowlby (92), attachment to the primary caregiver develops not earlier than after 8 weeks, with increasing differentiation in the second half of the infant’s first year and attachment measures are applied with 12–18 months. Because maternal postpartum psychiatric disorders occur within the infant’s first year of life, with an onset immediately until 4 weeks after birth, observational measures of attachment security need to be assessed at follow-up. The Ainsworth sensitivity and the NICHD-ECCN scales have been shown to be good predictors for measures of attachment security. Moreover, the latter two are freely available and open to use for everyone. For research, reliability testing is mandatory. Training to learn the manual of the CARE-Index takes about 8 days plus practice and reliability testing. Also, the EA Scales require 1 week with additional training by Biringen, too. Whereas the Ainsworth sensitivity and the NICHD scales are free for usage, the other two are subject to charges.

Another limitation is that the NICHD ECCN sample mostly stems from the middle class. For samples who are at specific risk to show highly dysfunctional parenting behaviors, however, it is necessary to use scales that particularly differentiate behavioral nuances at low levels of parenting competence and that allow to measure distinct impairments of specific aspects of parenting behaviors.

Furthermore, all methods vary with regard to the observational situation. Whereas the Ainsworth Maternal Sensitivity Scale and the EA Scales can be applied in different settings, the CARE-Index favors free-play situations, and the NICHD-ECCN Scale requires semistructured situations like diaper change or feeding situation or in the toddler form a manualized procedure. Also, the required recording length ranges from 3 min (CARE-Index) to 30 min (EA Scales). Regarding the special group of mentally ill mothers, the observational situation must be carefully chosen. It should be easy to implement during daily routine of several treatment institutions. Because those mothers are limited in their capacity to play freely with their children, it must not enforce the feeling of insufficiency.

Next, a crucial diagnostic criterion of postpartum depression is depressed mood and loss of interest, whereas arousal is increased in mothers with postpartum anxiety. Affect measures have been considered in all scales but only to a limited degree: Hostility or warmth is measured in the Ainsworth Maternal Sensitivity and EA scales. The NICHD-ECCN sensitivity composite score includes positive and negative regard of the child for youngest children, with changes in the negative affect scale in the toddler form.

Further considerations refer to how differentiated maternal behavior is measured, which is especially important with regard to mothers’ symptom comorbidity. Recent research finds that depression and anxiety during the first 8 weeks postpartum occur simultaneously in 13% of mothers (20). Symptoms of anxiety often come along with symptoms of compulsive behavior (93). Triple comorbidity of depression, anxiety, and posttraumatric stress disorder (PTSD) is relatively rare with a prevalence of 2% to 3% (94). Other comorbidities are not well studied, although they appear in clinical practice. Next to comorbid symptoms, stresses and strains as parenting or childhood stress, immigration history, and maternal vulnerable personality predict higher rates or symptoms (20). A main concern with the recent methods is that behavioral variance caused by comorbidity may not be represented sufficiently because all ratings use sum scores or general ratings. For instance, a mother with depression can partly be responsive and adequate but not prompt, whereas a psychotic mother might act responsive, prompt but not adequate. By using a sum scale of sensitivity, both mothers would receive low scores, but how can one differentiate behavior between these mothers regarding sensitivity? And how can such differences then be related to later child outcome? With the future goal, that is to describe the mother–child interaction in postpartum disorders with the full spectrum of comorbidities and risk factors, a separation of the concept of sensitivity into its original components described by Ainsworth is valuable.

To sum up, we propose an adaptation of the described methods to measure sensitivity to fully represent the spectrum of altered behavior in postpartum psychiatric disorders with all its comorbidities. Our intention was to develop a method that is applicable in a clinical routine setting (e.g., mother–baby unit) with reasonable effort for patients and the therapeutic team. At the same time, it should be usable for scientific purposes. Future scientific questions can then focus on how maternal caregiving behavior differs with regard to different diagnoses while taking into account the high number of comorbidities. It is also of special interest if other risk factors associated with postpartum psychiatric disorders are related to specific deficits, for example, parenting stress, traumatic experiences, or lack of social support. And finally, we aim to relate our findings to specific child outcomes to increase our understanding on symptom-specific effects of alterations in parenting behavior that are related to maternal mental illness.

The present paper describes the development of an adapted measure of maternal caregiving behavior for scientific and clinical use in mothers with postpartum psychiatric disorder. The adapted measure specifically considers the clinical setting, peculiarities of postpartum disorders, and a fine-grained description of maternal behavior. We then present a preliminary pilot implementing the adapted measure on 38 mothers admitted to a mother–baby unit and 35 healthy mothers.

## Method

### Ethics

This study was carried out in accordance with the Declaration of Helsinki and the permission and recommendations of the Ethics Committee of the Friedrich-Alexander University Erlangen Nuremberg and the Ethics Committee of Technical University Dresden (Ethics committee of the FA: 320_15 B, Ethics committee of the TU: EK450 22013). All participants gave written and informed consent. For all data concerning children, written informed consent was obtained from the parents.

### The Pilot Project: Video Screening for the Development of the Adapted Sensitivity Scale

#### Sample

Mentally ill mothers currently attending a video-based interaction therapy in a mother–baby day unit were asked to participate in the study. Video recordings were made on a voluntary basis. Treatment institutions were the Mother-Baby-Day-Unit of the Clinic of Psychiatry and Psychotherapy of the Paracelsus University Clinic Nuremberg Psychiatry in Nuremberg and the Mother-Baby-Day-Unit of the Clinic for Psychotherapy and Psychosomatics of the University Clinic Carl Gustav Carus at the Technical University in Dresden. In these units, mothers spent time from 8 to 16 o’clock each day for an average of 8 weeks, during which they receive a variety of interventions (e.g., group therapy, psychoeducation, personal counseling, and art therapy). A special focus lies in the improvement of mother–child interaction by providing sensitivity training, baby massage, and mother–baby bonding therapy. Video interaction therapy starts soon after the initial assessment with numerous sessions during the whole stay in treatment. In these sessions, mothers watch videotapes of themselves interacting with their infant and get feedback about positive sequences from their therapist. They are then supported by their therapist to enhance such behaviors and integrate them into daily life situations.

The total sample of mothers who agreed to participate in the current study consisted of 102 mothers with postpartum disorders (67 stemming from the Dresden Unit and 35 from the Nuremberg Unit) and of 38 healthy/nonclinical mothers. For mentally ill mothers, the diagnoses were depression, anxiety, compulsive disorder, PTSD, substance abuse {of medium extent [alcohol, tetrahydrocannabinol (THC), or nicotine during pregnancy and continuing]}, bipolar disorder, personality pathology, and/or psychosis (order according to frequency). More than half of the patients had more than one diagnosis.

Clincial diagnoses were based on clinical assessment according to International Statistical Classification of Diseases and Related Health Problems (ICD-10) criteria, video recordings were made by an interaction therapist, and questionnaires were assessed as self-ratings.

#### Observation of Mother–Child Interaction

Observation and analysis of mother–child interaction were used for the development of the adapted sensitivity scales. Videos were recorded in clinical routine for therapeutic use at the beginning of their therapy. Mothers were instructed to act as normal as during daily routine.

#### Description of the Adapted Sensitivity Scales for Observation

The method is based on the Ainsworth Maternal Sensitivity Scale (31) and the NICHD-ECCN Scale (80, 84, 95) and was adapted to the survey situation, children’s age, and peculiarities of interaction behavior in mothers with postpartum psychiatric disorders. Analysis of video-recorded interactions was done according to the manual attached (see **Supplementary Material**).

With respect to behavioral deficits in mentally ill mothers (as described above), the following scales were defined: caregiver’s ***responsiveness, promptness*** of reaction, ***appropriateness*** of the caregiver’s reaction, ***intrusiveness*** of the caregiver, and ***negative affect*** and ***positive affect*** toward the child.

The scale ***responsiveness*** refers to whether the mother reacts to the child’s signals. Here, given that the child sends a signal, overtly or subtle, the mother needs to signal back that she recognized it. For example, during diaper change, this can be the exchange of a look or comments on what is happening, when the child displays discomfort. ***Promptness*** is defined whether the response occurs immediately, not exceeding 3 s. Otherwise, the child may not relate its own action to the maternal reaction and experience himself as agent of this interaction.

The scale ***appropriateness*** measures whether the mother’s reaction fulfills or at least adequately attempts to fulfill the baby’s needs presented in the sequence. Generally spoken, this refers to security, warmth, and comfort. An appropriate reaction is well-rounded and children experiencing appropriate behavior are usually less stressed.

These three scales were chosen based on Ainsworth’s definition of sensitivity and the NICHD descriptions, whereas both of them use an overall score for all three types of behaviors referred to as “sensitivity.”

***Intrusiveness*** measures to what extent the caregiver respects the child’s autonomy and accepts its individuality. High intrusiveness indicates low respect of autonomy. Practically, this can often be observed when mothers unclothe their children in an uncomfortable but quick manner, instead of doing so with respect to the child’s reaction and by providing guidance through, for example, verbalization. Mothers with postpartum psychiatric disorders have difficulties here because they often seem to follow an adult-centered script and do not seem to be able to take over the child’s perspective.

***Negative affect*** is defined as the presence of active expressions of negative affect either verbally, facial, or by hostile actions. Because infants cannot differentiate whether they are the cause for maternal bad mood or whether there are other causes, all kinds of negative affect and hostility are rated. ***Positive affect***, in contrast, is mirrored in the caregiver’s joy within the interaction. These scales already existed in the NICHD-ECCN Scale and were modified with special regard to the altered affectivity in psychiatric disorders.

The method and the rating scheme are described at length in the manual at the appendix (compare **Supplementary Material**). We added examples that were found in the videos to the descriptions.

Scales and blinded videos were repeatedly discussed on lab meetings and with trained sensitivity observers. Other scales, as measuring maternal speech or body contact, were rejected during adaptation because they were not directly related to the concept of sensitivity, although many mothers also show deficits here.

In total, six scales were rated on a 9-point scale ranging from one to five with semisteps in between. Defined anchor points are 1, 2, 3, 4, and 5. Low values indicated low characteristics of the regarding scale, whereas high values go along with frequent observation of the behavior in the scale.

#### Coding of Mother–Child Interaction

Analyses were conducted by five postgraduate psychology students who participated in a seminar on “observational tools to assess baby’s signals” and underwent intense reliability training by experienced researchers. Reliability training included reading literature on sensitivity, discussing videos with mother–child interaction and doing pre-ratings to find misperceptions or positive or negative observation bias on a weekly basis for 6 months. They were trained with the help of the manual and an assessment sheet (see the Manual) analyzing the categories: Responsivity, promptness, appropriateness, intrusiveness, and negative and positive affect. Note: Observers who are familiar and reliable with other sensitivity scales should be able to use the method with less intense training.

Raters were blind to diagnoses. All five students were required to become highly reliable, with all κ > 0.85 before they were allowed to score the videos included in the study. According to McHugh (96), this indicates that interrater reliability is strong to perfect. *Post hoc* calculated interrater reliability (weighted kappa) was based on 17 double-coded video tapes after 6 months. Here, mean reliability was moderate, with κ = 0.73. All raters showed strong reliability on the scale intrusiveness, κ = 0.85, whereas on the scale, promptness reliability was lowest and just weak, with κ = 0.59. Reliability for the other scales were moderate with responsiveness, κ = 0.66; appropriateness, κ = 0.73; negative affect; κ = 0.76; and positive affect, κ = 0.80.

### First Statistical Analyses Comparing Data on Depressed and Anxious Mothers to a Healthy Group

#### Sample

For the present study, we examined a subset of the clinical sample including 31 mothers from the Nuremberg Unit and seven mothers from the Dresden Unit with postpartum depression and/or anxiety disorder. The clinical sample was recruited consecutively from 2016 to 2018. Infants were healthy as seen by Pediatrics from the hospital. There was a massive reduction of usable videos because during the pilot phase, criteria for the recordings of the videos for scientific usage have been developed.

Videos were excluded if they were <3 min in length (according to the standard CARE-Index procedure), recorded after the third week of admission, the patient presented with three clinical diagnoses on the basis that the underlying disorder was unclear (e.g., depression and anxiety and personality disorder and substance abuse), invisibility of important parts of the interaction, strong sound problems, or lack of questionnaire data.

In contrast, inclusion criteria were a good quality of the video, recording at the beginning of the therapy, and at least one of the diagnoses from the spectrum of depression, anxiety, or compulsive disorder.

The control group was acquired in the area of Erlangen Nuremberg, Germany, and videos were recorded during home visits. The control mothers were recruited *via* flyer or word of mouth and agreed to participate in a home visit, including the recording of a video and assessment of questionnaires. They were paid 20€ and received a small toy for the child.

All mothers were instructed to act as usual during daily routine. All videos included a semistandardized situation, where they changed the diapers of their infant or fed him or her. In addition, parents were asked to play with their infant without instruction.

#### Questionnaires

##### Brief Symptom Inventory

The Brief Symptom Inventory (BSI) (97) is a short form of the Symptom Checklist SCL-90-R (98) and aims to assess mental stress of participants within the last 7 days. Participants respond on a 5-step Likert Scale, ranging from 0 (“not at all”) to 4 (very heavy) how much they agree to a total of 53 items. The BSI includes nine scales assessing, for example, depression, anxiety, or psychotic experiences. Finally, the global score [Global Severity Index (GSI)] is calculated to assess general mental stress.

In addition, there are three global scores for general mental stress: GSI, intensity [(Positive Symptom Distress Index (PSDI)], and number of reported symptoms [positive symptom total (PST)].

Scores can be transformed into T scores with different norms for academic students or adults in general, as well as men and women. Here, we used T scores for women (nonstudent) to assess the global severity of mental illness. Clinically relevant GSIT scores are above ≥63 (99).

##### Edinburgh Postnatal Depression Scale

To assess postpartum symptoms of depression, we used the Edinburgh Postnatal Depression Scale (EPDS) (22). The screening instrument includes 10 items asking for the mood of the last 7 days of young mothers. Mothers had to answer on a 4-point Likert (0–3) Scale how much they agreed with each item. Scores were summed up, with 30 as the highest possible value. High values indicate strong symptoms. Mothers with scores of 13 or higher are regarded as likely to suffer from depression. Validation studies (100) reported good sensitivity (79%) and specificity (85%).

### Statistics

We used SPSS 25.0 for analysis. To examine differences between group variables, we used *t*-test. Intercorrelation analysis was done by Pearson correlation. Potentially influencing factors, as age of the child or mother, were also controlled by using Pearson correlation. Group comparisons regarding sensitivity were done with multivariate analysis of variance (ANOVA). All *post hoc* pairwise comparisons were performed using LSD. Significance level followed *p* ≤ 05*, *p* ≤ 0.01**, *p* ≤ 0.001***. **Tables** show means and standard deviations.

## Results

### Sample Descriptives

Mothers of both groups were at the same age in the beginning of the thirties and had the same, relatively high, educational background (**Table 1**). The sex of the children had a similar ratio in both groups. Children of mentally ill mothers were significantly younger than children of the control group. In the clinical group, the number of participants with at least one close relative (parents or grandparents) who suffered from mental disorders was twice as high as in the control group.

**Table 1:** Demographic data for healthy and clinical mothers.

	Healthy mothers (n = 35) Mean SD	Clinical mothers (n = 38)	
		Mean SD	Significant differences
Maternal age (years)	31.49 ± 4.27	30.02 ± 5.38	n.s.
Education with A Level (%)	62.85	55.26	n.s.
Age of child (weeks)	49.60 ± 25.22	27.75 ± 21.39	*t*(71) = 4.002, *p* = 0.000
Sex of child (boy:girl)	16:19	19:19	
PTs having relatives with psychiatric disorder	11	22	Χ^2^ (1, *n* = 73) = 5.152, *n* = 0.023
EPDS (sum)	5.77 ± 3.98	17.32 ± 5.55	*t*(70) = −10.097, *p* = 0.000
BSI (GSI)	46.97 ± 12.03	74.83 ± 7.72	*t*(71) = −11.763, *p* = 0.000

Values indicate means and standard deviations. PTs, participants; EPDS [sum], sum score of the Edinburgh Postnatal Depression Scale; BSI [GSI], Brief Symptom Inventory [Global Severity Index]; n.s., not significant.

Psychopathology was significantly higher in the clinical group as indicated by measures of the BSI (GSI) and EPDS. In the control group most participants (27 of 35) had scores below 10, while 5 participants had an EPDS score between 10 and 12, indicating a medium degree of postpartum depressive symptoms, and 3 of them had scores above 12 (clinical relevance). In contrast, in the clinical group, almost all mothers (36 of 38) had scores above 12, whereas 2 of them scored between 10 and 12.

### Group Comparisons

We used multivariate ANOVA to test if the group of healthy mothers differs in maternal sensitivity significantly from the clinical group. Although not significant (F_(6,66)_ = 1.690; *p* = 0.137), results indicate that the clinical group performs poorer in all subscales compared to the healthy control group (**Table 2**). Nonsignificance is typical when the dependent variables are highly correlated (**Table 4**). Further analysis underlines it: extracting promptness as a dependent factor from analysis leads to approaches of significance (F_(5,67)_ = 2.042; *p* = 0.084). However, univariate comparisons result in significant effects for each of the dependent measures. Healthy mothers are more responsive, more prompt, and more appropriate than mentally ill mothers. They respect more of the child’s autonomy, whereas clinical mothers are more intrusive. Negative affect is lower and positive affect is higher in the healthy group than in the clinical group. Whereas the group of healthy mothers perform in the upper range of the scale (range 3.87–4.33), clinical mothers perform still in a mediocre to upper range (range 3.12–3.88).

**Table 2:** Comparison of the sensitivity subscales for healthy and clinical mothers.

	Healthy mothers Mean SD	Clinical mothers	
		Mean SD	Significant differences
Responsivity	4.16 ± 0.73	3.67 ± 0.89	F_(1,71) =_ 6.431; *p* = 0.013
Promptness	4.01 ± 0.66	3.54 ± 0.93	F_(1,71) =_ 6.214; *p* = 0.015
Appropriateness	3.97 ± 0.86	3.32 ± 1.00	F_(1,71) =_ 8.942; *p* = 0.004
Intrusiveness	2.13 ± 0.83	2.88 ± 1.15	F_(1,71) =_ 10.203; *p* = 0.002
Negative affect	1.67 ± 0.69	2.12 ± 0.86	F_(1,71) =_ 5.984; *p* = 0.017
Positive affect	4.33 ± 0.86	3.85 ± 0.94	F_(1,71) =_ 5.003; *p* = 0.041

*Va*lues indicate means and standard deviation. P values indicate significance from multivariate testing.

### Factors Influencing Maternal Sensitivity

We tested for maternal and child age as possible parameters influencing sensitivity. Spearman correlation revealed no significance with these factors for none of the scales neither in the group of healthy nor clinical mothers (compare **Table 3**). For further testing if the difference of child age between the group still affected the results, we included it as a covariate into the analysis. Here, significance was reduced to a trend level in three scales, namely, responsivity (F_(1,70)_ = 2.820, *p* = 0.098), promptness (F_(1,70)_ = 3.594, *p* = 0.062), and negative affect (F_(1,70)_ = 3.816, *p* = 0.055).

**Table 3:** No correlation of maternal behavior with maternal or child age.

		Maternal age	Child age
	**Healthy mothers**		
1	Responsivity	−0.078	0.221
2	Promptness	−0.185	0.122
3	Appropriateness	−0.157	0.297
4	Intrusiveness	0.048	−0.154
5	Negative affect	−0.039	0.042
6	Positive affect	0.212	−0.045
	**Mothers with PPD**		
1	Responsivity	−0.074	0.137
2	Promptness	−0.043	0.084
3	Appropriateness	0.033	0.066
4	Intrusiveness	−0.100	−0.164
5	Hostility	−0.276	−0.320
6	Positive affect	0.230	0.017

### Intercorrelations Between the Scales of Sensitivity

As visible in **Table 4**, all scales correlate significantly with each other in the healthy control group (range from *r* = 0.934 to *r* = 0.446) and the clinical group (range from *r* = 0.955 to *r* = 0.620).

**Table 4:** Intercorrelations of maternal sensitivity in the control group (*n* = 35) (upper values, gray background) and the clinical group (*n* = 38) (lower values).

Clinical group		Healthy control group					
		1	2	3	4	5	6
1	Responsivity	1	0.934***	0.883***	−0.661***	−0.529***	0.506**
2	Promptness	0.955***	1	0.835***	−0.585***	−0.478**	0.460**
3	Appropriateness	0.826***	0.846***	1	−0.753***	−0.592***	0.594***
4	Intrusiveness	−0.664***	−0.620***	−0.824***	1	0.493**	−0.446**
5	Negative affect	−0.758***	−0.707***	−0.760***	0.742***	1	−0.837***
6	Positive affect	0.694***	0.659***	0.756***	−0.715***	−0.913***	1

Asterisks label significance (**p ≤ 0.005, ***p ≤ 0.001).

### Influence of Severity of Symptoms in Mental Illness on Sensitivity

One goal of the development of the adapted method is to analyze sensitivity with respect to specific symptoms of depression to better differentiate between groups of different mental disorders. In the group of healthy mothers, we found mothers who scored subclinical to moderate on depression or EPDS and/or BSI and were clinically conspicuous. We decided not to exclude them from earlier analysis because they were “healthy enough” not to be in treatment. However, in an additional analysis, we put them in a separate group, named “healthy with mild depressive symptoms” (*n* = 8). Moreover, we separated the group of clinical mothers. Whereas 16 mothers were diagnosed with depression only, 19 mothers had depression and anxiety disorder or compulsive thoughts comorbid. We excluded three mothers because their comorbid diagnosis appeared to raise a personality disorder. The healthy group without depressive symptoms consists of 27 mothers. It is obvious that, with increasing symptoms, EPDS and BSI scores rise (**Table 5**).

**Table 5:** Comparisons of the sensitivity subscales for groups with different degrees of depressive symptoms, ranging from no symptoms to comorbidities.

	Healthy mothers			Clinical mothers			
	No depression *n* = 27	Mild depression		Depression		Depression + anxiety	
		*n* = 8	*p*	*N* = 16	*p*	*N* = 19	*p*
EPDS	3.96 ± 2.31	11.87 ± 1.35	0.000	14.87 ± 5.43	0.000	18.61 ± 5.03	0.000
BSI GSI	43.03 ± 7.83	61.50 ± 12.75	0.013	72.81 ± 7.47	0.000	76.89 ± 3.98	0.000
Responsivity	4.24 ± 0.69	3.87 ± 0.79	n.s.	3.65 ± 0.97	0.029	3.73 ± 0.88	0.047
Promptness	4.05 ± 0.62	3.87 ± 0.79	n.s.	3.53 ± 1.04	0.050	3.57 ± 0.91	n.s.
Appropriateness	4.07 ± 0.80	3.62 ± 0.99	n.s.	3.21 ± 1.04	0.006	3.42 ± 1.03	0.025
Intrusiveness	2.02 ± 0.82	2.50 ± 0.75	n.s.	2.88 ± 1.27	0.008	2.79 ± 1.04	0.012
Negative affect	1.67 ± 0.65	1.69 ± 0.84	n.s.	2.00 ± 0.98	n.s.	2.16 ± 0.74	0.048
Positive affect	4.33 ± 0.82	4.31 ± 1.03	n.s.	4.00 ± 1.01	n.s.	3.78 ± 0.85	0.040

Mean and standard deviation. p values belong always to comparisons from the group left to them with healthy mothers without depression.

n.s., not significant. See ref. (24).

EPDS, Edinburgh Postnatal Depression Scale; BSI GSI, brief symptom inventory, global severity index.

We used multivariate analysis to test whether the sensitivity scales differ significantly between the four groups (healthy vs. mild symptoms vs. clinical with depression vs. clinical with depression and comorbidities). Not significantly, but descriptively, with increasing severity, sensitivity gets worse. Healthy but mildly depressed mothers have lower values in sensitivity but not in their affect. Clinically burdened mothers differ significantly in their sensitivity pattern from that of healthy mothers. Whereas mothers with depression respond less, are less prompt, less appropriate, and more intrusive, their affect does not differ significantly from that of healthy mothers. Mothers with additional diagnoses of anxiety are less responsive, less appropriate, and more intrusive and show more negative and less positive affect but are not significantly less prompt.

## Discussion

### General Discussion

The present study aimed at adapting observational methods assessing sensitivity in caregivers to a group of mothers with postpartum mental disorders. Therefore, we screened 102 videos of mentally ill mothers interacting with their infants to identify specific parenting behaviors associated with different postpartum mental disorders. These specific behaviors are described in the manual, which can be found in the supplements. With respect to the main deficits of maternal caregiving behavior, we combined different measurement scales for sensitivity. More precisely, with special regard to attachment research, the Ainsworth’s Maternal Sensitivity Scale (31) and the NICHD Scale were chosen as a basis for the present method while integrating aspects taken from the EA Scales, the CARE-Index, and from a range of other observational tools focusing on parental behavior (76, 79). This procedure resulted in the examination of responsiveness, promptness, appropriateness, intrusiveness, negative affect, and positive affect. The compilation of these final scales is adapted to the special group of mothers with postpartum disorders because it considers peculiarities regarding the onset of illness, child age, and disorder-related behavior.

In a second step, we used the adapted scales for a preliminary analysis aiming to investigate whether the method is applicable in a clinical group. Therefore, we compared caregiving behavior in a group of mothers at the beginning of treatment in a psychiatric mother–baby unit to that of a group of healthy mothers. Results revealed that mentally ill mothers have difficulties in all aspects of sensitivity, intrusiveness, and affect. Mothers with postpartum psychiatric disorders were found to be less responsive, respond slower, less adequate and to be more intrusive and show more negative and less positive affect toward their children. These findings are not surprising because it has been repeatedly described that postpartum depression can result in tremendous problems regarding mother–child interactions [e.g., Refs. (25, 26, 30, 55, 98)].

A second aim was to see whether assessing different aspects of sensitivity separately would help identify typical behavior patterns of mothers with mental disorders dependent on their diagnosis or other risk factors for altered mother–child interaction. Here, we found that the severity of symptoms is an important factor influencing maternal sensitivity in the present sample. Even in the healthy group, we observed that mild depressive symptoms already reduce sensitivity, however, the effect was rather small. This is in line with recent research showing that subclinical depressive symptoms may increase hostility and negative affect (51) and reduce maternal sensitivity (101). Moreover, we separated the clinical group into those with depression only and those who have an additional diagnosis for anxiety disorder because postpartum depression and postpartum anxiety are frequently comorbid (102, 103). Comparing results to the healthy control group revealed deficits in both groups of mothers with mental illness, but we found different behavioral patterns: In contrast to healthy mothers, those with depression only scored significantly lower on all scales of sensitivity (responsiveness, promptness, appropriateness) and intrusiveness; they did not differ significantly on the affect scales, although values are lower than those in the healthy group. In comparison, mothers with comorbidities of depression and anxiety scored, apart from promptness, significantly lower on all subscales, indicating general deficits in mother–child interaction including own emotion regulation.

The difficulties with sensitivity and intrusiveness in depressive-only mothers have repeatedly been found in earlier studies. Responsiveness, the perception and reaction to a child’s signal, has repeatedly been shown to be lower in postpartum depression (25, 32–35). Moreover, maternal responses are less contingent (25) and less appropriate (33). Intrusiveness and withdrawal are reported to appear inconsistently (16, 25), whereas in our study, depressed mothers generally tend to show less respect for child autonomy. Without the investigation of a behavioral pattern, the lack of responsiveness alone may lead to the assumption that signals are not perceived, likely as a result from a cognitive deficit. In combination with measures of lower appropriateness and higher intrusiveness, it can be assumed that mothers ignore their children purposely, followed by a parent-centered response inappropriate to the child’s needs. The underlying cognitive process behind this behavior remains unmeasurable, but it seems as if intuitive behavior is disturbed in depressed mothers (97) and that they rather follow a script or external recommendations during interaction. Also, depression worsens the ability to manage changes between affective states, and it is more difficult to adjust to their child’s affective states and needs sensitively (49, 50). Interestingly, in our study, maternal depression did not significantly alter affect during mother–child interactions in mothers with depression only, although depressive mood is central in depression. In contrast, Cohn and colleagues (104) argue that negative affect is increased four times in depressed mothers. Also, prior work found mothers with former or postpartum depression to show more negative behaviors as criticizing or destructive reactions and fewer positive behaviors, such as praise or constructive guidance during interaction sequences (105, 106). One reason why we did not find this could be that, in the present sample, the variance in the affect scale is relatively increased in clinical mothers as compared to healthy mothers. This indicates that some of them are well capable of positive interaction with their infants, whereas others are not. Laucht et al. (107) suggest, that, because of some well-functioning, self-control mechanisms, many depressive mothers are capable of acting as nondepressed in contact with their children, warranting a positive child development (107).

As general explanation, the disturbances in altered mother–child interaction are caused or accompanied by altered brain activation. Mothers with postpartum depression have shown weaker activation of reward and motivation areas, such as the thalamus, nucleus accumbens, caudate, and key emotion regulation areas, such as the lateral orbitofrontal cortex (108). Lower responsiveness may be the result when motivation is lowered to increase contentment and infant stimuli do not feel as rewarding for some of them as for healthy mothers.

In our subsample, mothers with depression and anxiety comorbid presented another behavioral pattern. This group showed deficits in all scales, except promptness, which may represent the increase of impairment caused by the double diagnosis.

Earlier findings on maternal anxiety describe mothers as less responsive and withdrawing from interaction, also in trait anxiety of healthy mothers after birth [e.g., Refs. (36, 37)]. More precisely, anxious mothers behave less appropriate (39) and are more exaggerating or overprotective (26, 38). This exaggeration or overprotection, probably caused by the typical higher arousal in anxiety, is mirrored in the “promptness” scale. In our study, we found mothers with comorbid anxiety to have somewhat lowered scores on this scale; however, they did not significantly differ from those of the healthy group. In the face of comorbid anxiety, promptness may be a sign of higher alertness to threatening keys (such as unfamiliar baby signals combined with low maternal self-efficacy). In our, as well as in recent, studies, highly anxious mothers present more intrusive behaviors compared to mothers with low anxiety levels [e.g., Refs. (36, 37, 43)] and support less autonomy (46). They interfere with and try to control the child’s behavior (37, 38, 41), often with the objective to be overprotective (53, 109).

The finding that responsiveness, appropriateness, and intrusiveness are worsened in comorbidly ill mothers may reflect a commonality with depressed mothers’ behavior, namely, that they act more parent-centered and less intuitive. The constant alertness reflected in the promptness scale could give a hint to their inner conflict between “waiting for signals” and “not being able to react flexible.” This conflict is also visible on the affect scale because they show less cheerful play and more negative affect mimically and verbally, more distressed behavior, or emotional flattening. Negative affect in maternal anxiety has been observed through stress and sorrowful behavior (53), increased criticism, and less satisfaction of the child’s behavior (54, 55). In our study, we observed lowering of positive affect in depressed and anxious mothers in contrast to some other studies (26, 58–60). Our finding is in line with those studies describing depressed and anxious mothers as generally less warm and positive in contact with their children, indicated by less smiling and positive gestures (46, 54, 56, 57). The lack of joy in the postpartum phase might also have its cause in altered brain processes, namely, that higher levels of anxiety in relation to parenting correlated with reduced activation in the substantia nigra, a region implicated in reward prediction and processing (110). When a child’s smile feels less rewarding, experience of joy is probably reduced, too. Consequently, intentions to make the child laugh may be lacking.

In sum, the presented scales successfully identify specific deficits in mother–child interaction of mothers with postpartum psychiatric disorders. It further shows that these deficits differ depending on the diagnosis or comorbidity of mothers and thus provide simplifications for tailored therapeutic intervention. Although for depressed-only mothers, a training on improving appropriate reactions and on understanding of infant’s signals and needs might be sufficient, mothers with depression and anxiety comorbidity may need further support in their own emotion regulation. Our method is very detailed, but it is still closely related to the concept of sensitivity. Therefore, it is suited for the investigation of maternal behavior as a predictor of later attachment security.

### Limitations

Results of our study are based on a group of healthy mothers and a subgroup of mothers admitted in a psychiatric/psychosomatic Mother-Baby-Day-Unit Nuremberg or Dresden. Healthy and nonhealthy mothers did not differ in their educational degree or age. One limitation was that the children of clinical mothers were younger than those of the control group because of a change of admission requirements in the hospital while the study was still ongoing. However, there was no correlation of the scales with child’s age.

Behavioral assessment was located differently for clinical and healthy mothers that may have caused different levels of stress or comfort during observation.

Another limitation of the study is the rather small sample size in the group with comorbidities with respect to the fact that they are diagnosed with different and/or several anxiety disorders. Anxiety disorders manifest in a wide range: generalized anxiety disorder, OCD, panic disorder, and birth-related PTSD. In the postpartum period, sometimes the severity and symptoms do not rise to the level of an anxiety disorder diagnosis (e.g., hypervigilant concerns and attention for the baby, extreme lability, constant worry) but nevertheless can cause significant distress and disturb mother–child interactions (93, 111). Creating further, more clearly defined subgroups would have yielded in too small sample sizes. So, conclusions for different diagnoses of anxiety on sensitivity must be postponed to future studies. Still, the investigation of subgroups and comorbidities remains an important goal for the present method.

### Further Thoughts on the Use of the Method in Postpartum Psychiatric Disorders

#### Intercorrelations

During development of the method, it was noted repeatedly that “responsiveness” and “promptness” correlate highly significantly and were difficult to separate. We thought about combining these two scales again as the NICHD-ECCN Scale does but decided to keep them separate. First, we found “promptness” to differentiate between depressed-only and depressed-anxious mothers, as presented above. Second, during scale adaptation, we often found single videos (psychosis or very severe depression), where the difference was more obvious as responsiveness was very delayed. Third, all scales correlate with each other what is typical in this research sector. All EA Scales (86) correlate with each other, and in the Ainsworth Maternal Sensitivity Scale, the author describes the scale “sensitivity” as a key variable correlating with the other scales. The positive and negative affect scales correlated very high, too, especially in the group of mothers with mental illness. This is likely to represent the emotional flattening of mothers with depression.

#### Scaling

A related discussion concerns the scaling of the subunits. A rank of 3 (the mean) relates to a mother who behaves a bit more often with good caregiving skills than bad. Healthy mothers rank relatively high (around 4 from 5). Our present clinical subsample ranges from 5 to 2; only in training videos did we find ratings of 1. Why is this so and should we have adapted the scaling? The scale is not conceptualized to separate very good from perfect maternal behavior. With a rank of 4 or 5, we can assume that the behavior in the observed sequence was ideal. It must be noted that the situation was short had only little instruction. A healthy mother often knows how to perform ideally and can act so for a certain amount of time in stress-free situations. Mentally ill mothers are not necessarily “disturbed” in their sensitivity but limited in their capacities (107) and can partly behave normal. Moreover, although the group presented here is severely impaired, the therapeutic staff decided that the child’s well-being is not critically endangered. Otherwise, they must have separated mother and child and decided to treat the mother as inpatient first. We believe that, with more demanding observational settings or other types of mental disorder as psychosis, scores will be lower. Methods of stress reduction or increase can change maternal performance, leading to higher variance between mothers.

#### Suggested Video Length and Observational Situation

In hospital routine, we implemented video observations with a length of 10 min displaying diaper-changing situations and free play. Especially in the group of mentally ill mothers, videos from the beginning of our studies were strikingly shorter. Whereas the CARE-index requires 3 min of observation of mother–child interaction only, the EA Scales (86) recommend analyzing much longer observations, up to 2 h. Both observations pursue different goals, namely, to decide about intervention with the CARE-Index (91) while extracting information about attachment measures with the EA Scales. Therefore, different recording times are reasonable. In our study, we recommend recording times around 10 min and include a semistructured situation and free play. Because attention span is reduced in depression, video observations should reveal deficits in maternal behavior quickly (112, 113), although many are also capable of sustaining normal behavior for a while. To increase motivation for participation and compliance for therapy and science, stress should be as low as possible during recording.

According to our clinical observations, mentally ill mothers seem to lack ideas for interactive play that may evoke feelings of insufficiency in the face of a given play task. If in future studies, higher differences between the groups are favored, stress in observation situation can be increased by using more difficult tasks or observe mothers with siblings (114). Not only attention itself but also divided attention is reduced in depression (112), what should also affect mother–child interaction in more demanding situations. In this sense, it has been shown that contact to a stranger reduces responsivity to child’s signals in mothers with social phobia (115).

### Future Directions

The present adapted method is useful to measure sensitivity in postpartum psychiatric disorders. Moreover, it should allow testing for relationships to attachment qualities with later strange situation procedures. Still, the next phase of research includes validation work with other observational tools and measures of attachment. Moreover, an extension of the sample with other diagnoses as psychosis and OCDs is planned. Analyses for further understanding of the disorder and its relation to maternal behavior and then child outcome are prospects. Of further importance in the group of mothers with postpartum psychiatric disorders is the interplay of genetic and caregiving influences in the prediction of attachment and further development of the child. It could be shown that higher maternal sensitivity can be a buffer for disorganized attachment in cases of genetic risk (116).

To describe the full spectrum of deficits in mother–child interaction, risk and protection factors for altered sensitivity postpartum psychiatric disorders should be assessed. Own childhood experiences are a crucial factor for the quality of sensitivity, with adverse experiences leading to less sensitivity, whereas positive experiences make warm and sensitive parenting more likely (117, 118). Moreover, experiences of emotional or physical abuse during childhood are predictors for hostile behavior toward her own child (119), as well as for postpartum maternal depression (120). Lack of social support is related to lower sensitivity and responsiveness toward her own children (121, 122) and a key factor for the development of depression (12). Socioeconomic pressure, as low income or unemployment, leads to lower quality in interaction (123) and increases depressive symptoms (12). In contrast, higher education and maternal employment are related to more sensitivity and less control of children (124). For future studies it may be interesting to study those risk and protection factors that play a role in the development of mental health for both parent and child.

## Ethics Statement

This study was carried out in accordance with the Declaration of Helsinki and the permission and recommendations of the Ethics Committee of the Friedrich-Alexander University Erlangen Nuremberg and the Ethics Committee of Technical University Dresden (Ethics committee of the FA: 320_15 B, Ethics committee of the TU: EK450 22013). All participants gave written and informed consent. For all data concerning children, written informed consent was obtained from the parents.

## Author Contributions

CH and MG designed and conducted the study, created the manual, and wrote the manuscript. SG was involved in study design, setup of the manual, assessment of control group, and editing the manuscript. JJ-H and SS were involved in study design and assessment of the clinical group and editing the manuscript. JF worked on the manual and the introduction. GS was involved in study design, edits on the manual, statistics and edits on the manuscript.

## Conflict of Interest Statement

The authors declare that the research was conducted in the absence of any commercial or financial relationships that could be construed as a potential conflict of interest.
