# Supplementary material for: Mothers With Postpartum Psychiatric Disorders: Proposal for an Adapted Method to Assess Maternal Sensitivity in Interaction With the Child
Source: Front Psychiatry. 2019 Jul 22;10:471. doi: 10.3389/fpsyt.2019.00471 (PMC6661973; doi:10.3389/fpsyt.2019.00471)
Supplement: Supplementary file 1 [file DataSheet_1.docx]

**Mothers with postpartum psychiatric disorders: An adapted method to assess maternal sensitivity in interaction with the child**

MANUAL

**Authors**

**Heinisch C & Galeris M, Gabler S, Simen S, Junge-Hoffmeister J, Fößel J, Spangler G**

| **1. Responsiveness.** | |
| --- | --- |
| Responsiveness is described as the perception of a baby’s signal as well as the caregiver’s reaction to the signal. A child’s signal is defined as an explicit attempt to communicate with the caregiver (e.g. crying or screaming) as well as subtle signals such as turning away its head. A *new signal* begins when after one signal (e.g. smiling towards the caregiver) another signal (e.g. holding a toy towards the caregiver) is sent or when the child sends the same signal for a second time after a break. This is the case if the child is briefly occupied with something else or the caregiver reacted to the first signal. Only the behaviors that are an attempt to communicate with the caregiver are counted as signals (e.g. turning away eye contact; *not:* child playing with a vibrating bell). | |
| Examples for a child’s signal could be:  *smiling (towards the caregiver) - making sounds - seeking eye contact - stretching out the arms - touching the caregiver/ holding on to the caregiver - pointing towards something in the room/ trying to show the caregiver something - screaming/crying/whimpering/whining*  *turning away the glance or closing the eyes due to excessive demand*  Examples of responsive behaviors are:  *making eye contact with the baby - interrupting the current activity - changing of the caregiver’s facial expression and gesture (e.g. frowning, startling…) - verbal affirmation of the perception - smiling at the baby - touching/ stroking the baby - talking to the baby (e.g. calmingly) - nonverbal communication (e.g. grimacing) - taking sth. from the child/ passing it sth. (e.g. to play) - changing the baby’s position - lifting the baby up* | |
| ***5. Highly responsive.*** | A highly responsive caregiver doesn’t have any problems recognizing explicit and distinct signals of his/ her baby as well as signals that are more subtle. He/she hardly ever misses a signal, acknowledges the signal and reacts consistently. A highly responsive caregiver can switch his/her attention between chores and his/her baby easily. |
| ***4. Mostly responsive.*** | A caregiver that acts responsive most of the time recognizes the majority of the baby’s signals, acknowledges and reacts to them. There can be times when the caregiver misses (subtle) signals or doesn’t react, but those are isolated incidents. Concerning explicit signals, the caregiver reacts always and consistently. A mostly responsive caregiver can mostly switch his/her attention between his/her chores and his/her baby. |
| ***3. Inconsistently responsive.*** | Although the caregiver reacts highly responsive to his/her baby’s signals at times, there are times in which he/she doesn’t recognize and/or react to his/her baby’s signals. This inconsistency can be explained by that the caregiver only perceives and reacts to certain kind of signals while always missing others. Inconsistently responsive caregivers often struggle dividing their attention between their own chores and their baby. Overall an inconsistently responsive caregiver is responsive more times than unresponsive and always perceives obvious signals of her baby. |
| ***2. Mostly unresponsive.*** | A mostly unresponsive caregiver doesn’t perceive or react to most of his/her baby’s signals. He/she struggles to divide his/her attention between his/her own activities and the baby or he/she is too involved in his/her own needs to be responsive to his/her baby’s needs. There can be times when he/she perceives the signals and/or reacts to them. This is mostly the case if a signal is obvious and persistent or if both share the same needs. |
| ***1. Very unresponsive.*** | The caregiver barely perceives any of his/her baby’s signals and/or decides not to react to them. He/she barely manages to divide his/her attention between his/her own chores or needs and his/her baby. A very unresponsive caregiver doesn’t react to facial expression, gesture or verbally to his/her baby’s signals. A reaction can only be noticed when the baby’s signal is very pressing, intense and persistent. |

| **2. Promptness.** | |
| --- | --- |
| A prompt or quick reaction is defined as a reaction occurring within three seconds of the baby’s signal. A prompt reaction gives the baby a feeling of consistency and effectivity of its own actions. If a reaction occurs very delayed or fails to appear at all, the baby is not able to make that experience. | |
| Examples of promptness  A caregiver for example who perceives a baby’s signal while throwing the diaper into the trash or finishing an urgent act is still rated as promptly if this happens within the time frame of about 3 seconds. | |
| ***5. Very prompt reaction.*** | The caregiver nearly always reacts to both, subtle as well as explicit signals, in a contingent and quick way. |
| ***4. Mostly prompt reaction.*** | The caregiver reacts to his/her baby’s signals most of the time in a prompt and contingent way. There are few times when the caregiver doesn’t react to subtle signals or the reaction is delayed. |
| ***3. Inconsistently prompt reaction.*** | The caregiver reacts to some signals contingently and quickly to others highly delayed or not at all. However, prompt reactions outbalance times of passivity or delayed reactions. |
| ***2. Mostly delayed or missing reaction.*** | The caregiver doesn’t react to a majority of the baby’s signals or reacts in a delayed way. However, there can be isolated moments in which the caregiver reacts prompt and consistently. |
| ***1. Very delayed and missing reaction.*** | The caregiver barely reacts to any of his/her baby’s signals. If he/she reacts sporadically the reaction is very delayed. |

| **3. Appropriateness of reaction.** | |
| --- | --- |
| A reaction is assessed as an appropriate reaction if the caregiver’s reaction fits the baby’s needs. Depending on the situation different approaches can be appropriate. If the baby only displays small signs of agitation it can be appropriate for the caregiver to talk calmly to the baby. If the distress is extended holding or stroking the baby can be appropriate. Nevertheless, there can’t be a high rating for appropriateness if the cause for calming down is not the result of an appropriate reaction, e.g. the baby calmed itself down. A high rating will not be given if the caregiver didn’t react or the reaction was too weak even if the child is calm in the end. An appropriate reaction evoked by non-distress signals of the baby consists of the caregivers response depending on the situation. A reaction can be assessed as appropriate even if the baby doesn’t calm down if it’s apparent that the caregiver did everything in order to calm the baby, but the child could not be satisfied due to external circumstances (e.g. pain, noise ...). | |
| Examples of appropriateness:  *If the signal was a simple (baby’s) eye contact an appropriate reaction could be to reciprocate the eye contact and smile at the baby. If the signal was the baby blabbering an appropriate response could be to speak along with the baby, comment it affectionately. If the baby cries hugging could be appropriate.* | |
| ***5. Highly appropriate reaction.*** | The caregiver almost always reacts to the baby’s needs. He/she knows when his/her baby “asks” for interaction or not and reacts accordingly (in situations when the baby is restless as well as in simple interactions). |
| ***4. Mostly appropriate.*** | The caregiver can interpret explicit signals and reacts appropriately to them. Sometimes the caregiver has difficulties interpreting non-verbal or subtle signals and accordingly reacts not in a completely appropriate way. |
| ***3. Inconsistently appropriate.*** | The caregiver can react to some signals in an appropriate way. His/her behavior is not steady. He/she can interpret and react (to) his/her baby’s signals correctly, but only sometimes does so. |
| ***2. Mostly inappropriate*** | The caregiver either does not understand what his/her baby wants from him/her and therefore reacts inappropriately, or he/she understands the baby’s intention but still doesn’t react accordingly. However, the inappropriateness is not completely consistent. If the baby’s needs agree with the caregiver’s needs or mood the caregiver may react appropriately. |
| ***1. Very inappropriate.*** | The caregiver shows no sign of understanding the baby’s needs with his/her reaction. He/she only satisfies his/her own needs with his/her reactions and doesn’t respond to the baby’s needs. |

| **4. Intrusiveness vs. Respect for the child’s autonomy /Non-Intrusiveness** | |
| --- | --- |
| This scale measures the degree to which the caregiver can recognize and respect the significance of the child’s autonomy, motives and perspectives. Intrusive caregivers are guided by their own desires and impose their opinions on the child even if the child signals that it wants to change the activity or needs a different speed of interaction.  A caregiver with low ratings on the other hand acknowledges the child’s desires as an important part of its individual identity. A non-intrusive caregiver doesn’t question the child’s right of its own needs and tries to rather guide its activities than to control them. Usually, a non-intrusive interaction is child-centered and not adult-centered. Also, he/she tries to integrate his/her own desires, moods and chores with the child’s desires, moods and current activities, so that a supportive interaction develops. A non-intrusive caregiver doesn’t disrupt his/her child’s activities. He/she waits with his/her intervention until a neutral pause occurs in the child’s activity. He/she knows how to direct the child’s activity step by step from the current activity to the activity he/she wishes it to do in a playful and mediating way.  When the caregiver grants autonomy to the child because of his/her own passivity are not scored as highly non-intrusive. Caregiver’s behaviors that serve security and protect the child are not rated as intrusive.  It must be noted the caregiver’s behavior needs to be evaluated independently of the child’s reaction (e.g. child laughs as the ball is taken away, because it thinks it’s a game). | |
| Examples of intrusiveness:  *Behaviors to which the child reacts with negative affectivity, turns away or fights back while the caregiver continues his/her behavior - continuously stimulation (physically and/or verbally); overwhelming with toys - not permitting the child to co-determine the pace or focus of the game/ play/ changing or feeding situation - taking away objects (e.g. toys, diapers) when the child seems to be still interested in them - not permitting the child to touch toys/clothes it grabs for - insisting on the child doing sth. that it doesn’t show interest in - not permitting the child to make decisions - manipulating the child’s body in a rough/ fitful way - impairing the child’s movement physically (rough/ unnecessarily) (e.g. pressing both legs roughly to the mat while changing) - verbally enforcing expressions (not explaining the further proceeding to the child)* | |
| ***5. Highly intrusive*.** | The interaction is characterized by a caregiver completely controlling his/her child. He/she rarely permits the child to navigate its activities alone. The caregiver grants the child little autonomy and negates the child’s experiences fundamentally. The caregiver denies and/or ignores the childÄs need for autonomy completely. |
| ***4. Mostly intrusive*.** | The pace of the interaction is mostly controlled by the caregiver and his/her behavior is poorly synchronized with the child’s signals. The intrusive behavior even continues when the child displays defensive and/or avoiding behaviors. The caregiver doesn’t deny the child’s individuality completely. However, he/she expresses clearly that the child’s intentions have less importance than to his/her own. He/She often intrudes in the child’s activities, so that the child has limited possibilities to do something by itself. |
| ***3. Inconsistently intrusive.*** | Caregivers who either frequently express weak signs or some clear signs of intrusiveness are scored as inconsistent intrusive. The intrusive behavior might occur inconsistently. Even if the inconsistently intrusive caregivers don’t deny the child’s independent identity, they do little to support it actively. Instead they may doubt the appropriateness of the child’s intention or intrude repeatedly in the child’s activity. |
| ***2. Mostly non-intrusive.*** | Single signs of intrusiveness can be present; however, they are not typical for the interaction. The caregiver might initiate interactions or present the child with proposals, that are occasionally unwanted. Sometimes the caregiver continues his/her activity although the child reacts defensively, but he/she doesn't expand the activity nor increases its intensity. |
| ***1. Highly non-intrusive.*** | The interaction is clearly child-centered. The caregiver encourages the child to express its intentions, negotiates the play interaction or allows the child to co-determine the pace during a changing situation. The non-intrusive caregiver allows and supports the child’s autonomy, while also continuing his/her own desires and individuality. By doing so he/she acknowledges the child’s perspective and never acts intrusively even when carrying through his/her own intentions. |

| **5. Hostility vs. Lack of negative affect towards the child** | |
| --- | --- |
| This scale rates whether a caregiver displays signs of negative emotionality or hostility towards the child. A caregiver with low ratings of negative affect doesn’t expresses signs of negative affectivity towards the child neither verbally nor non-verbally (e.g. facial expressions). The scale includes the quality as well as the quantity of the negative emotions towards the child. Since the baby usually cannot distinguish if he/she is the source of the anger/negative emotion, the caregiver’s negativity is rated as a general appearance. Caregivers with low negative affect, but also a low display of other emotional affects range in the lower scale. | |
| Examples of hostility or negative caregiver affectivity towards the child can be:  *disapproval - tense posture - negative tone when correcting the child – abruptness - tense face muscles/ strained expression – harshness - threatening the child/punishing the child without explanation - coarseness when wiping out the face, changing the diapers or burping the child - belittling the child verbally (through taunts/ teasing)* | |
| ***5. Strong negative affectivity towards the child.*** | These caregivers express the negative affectivity they feel for their child in a strong way or display a moderate level of a negative view on their child permanently. The parent-child-interaction is nearly exclusively determined by the caregiver’s negative affectivity. |
| ***4. Predominant negative affectivity towards the child.*** | Permanent signs of a negative view on the child in low intensity can be observed. Alternatively, some indicators of a negative view in a high intensity can be identified. During their interaction the negative outweighs the positive. Taunts frequently fall into this category, too. |
| ***3. Medium negative affectivity towards the child.*** | The caregiver displays some signs of negative affectivity towards the child or a negative view on the child or an especially intense expression of the negative view. It is also possible that the caregiver shows a mix of negative and positive affectivity towards the child. |
| ***2. Slightly negative affectivity towards the child.*** | Caregivers display little negative affectivity towards the child or minimal signs of a negative consideration. Though some small indicators of negative affectivity can be observed, the intensity of the negative expression is moderate and/or low. |
| ***1. No negative affectivity towards the child*** | This rating is given to caregivers who express no signs of negative affectivity towards the child neither verbally, nor through facial expressions or gestures. No indication of anger, suspicion, frustration, impatience, loathing, general antipathy or any other indicator of a negative view on the child can be found with those caregivers which is expressed in their face or tone. |

| **6. Positive affectivity towards the child / positive view on the child.** | |
| --- | --- |
| Positive affectivity towards the child is clearly expressed when the caregiver listens to and/or, attentively looks after the child. They have loving eye-contact when talking to him/her and/or show physical contact. The caregiver treats the baby respectfully, and playfully.  Mothers with low positive affect but low general affectivity, scale in the lower range. | |
| Examples of positive affectivity are:  *speaking in a warm tone - hugs or other expressions of physical affection - expressive face – smiling - laughing with the child - enthusiasm about the child - praising the child - general enjoyment of the child* | |
| ***5. High positive affectivity towards the child.*** | This rating is given to caregivers who are extraordinarily positive in their facial and vocal expression as well as in their complete behavior. The caregiver displays a spectrum of consistent positive expression towards the child. It is evident that he/she enjoys being with the child. |
| ***4. Mostly positive affectivity towards the child.*** | This caregiver predominantly displays a positive view on the child, although it is not completely consistent or strong. |
| ***3. Medium positive affectivity towards the child.*** | This caregiver displays some strong signs of a positive view which are however less frequent or intense compared to caregivers with higher rating. Caregivers with this rating either show inconsistent positive affectivity or a negative view on the child mixed with positive affectivity. |
| ***2. Little positive affectivity towards the child.*** | This rating is given to caregivers who show isolated or weak signs of positive affectivity. The intensity as well as the frequency of the positive affectivity is low. |
| ***1. No positive affectivity towards the child.*** | The number of positive signals is so low that it is a cause for concern. This rating is also assigned when positive affectivity (laughing, smiling) is inappropriate in observed situations or seems to be clearly fake. Taunting also fall into this category. |

**Assessment – Observation: Project Code: Date: Rater:**

1. **Responsivity, Promptness, Appropriateness**

| **Time** | **Nr.** | **Child** | | | **Caregiver** | | | | | | | | | **child** | |
| --- | --- | --- | --- | --- | --- | --- | --- | --- | --- | --- | --- | --- | --- | --- | --- |
|  |  | Description of the signal | Affekt | | Decription of the reaction | Affect | | Responsive | | Prompt | | Appropriate | | Affect | |
|  |  |  | + | - |  | + | - | yes | no | < 3 | > 3 | yes | no | + | - |
|  | 1 |  |  |  |  |  |  |  |  |  |  |  |  |  |  |
|  | 2 |  |  |  |  |  |  |  |  |  |  |  |  |  |  |
|  | 3 |  |  |  |  |  |  |  |  |  |  |  |  |  |  |
|  | 4 |  |  |  |  |  |  |  |  |  |  |  |  |  |  |
|  | 5 |  |  |  |  |  |  |  |  |  |  |  |  |  |  |
|  | 6 |  |  |  |  |  |  |  |  |  |  |  |  |  |  |
|  | 7 |  |  |  |  |  |  |  |  |  |  |  |  |  |  |
|  | 8 |  |  |  |  |  |  |  |  |  |  |  |  |  |  |
|  | 9 |  |  |  |  |  |  |  |  |  |  |  |  |  |  |
|  | 10 |  |  |  |  |  |  |  |  |  |  |  |  |  |  |

**Assessment – Observation: Project Code: Date: Rater:**

1. **Intrusiveness, Positive Affect, Negative Affect**

| **Time** | **Nr.** | **Child** | | | **Caregiver** | | | | | **Child** | |
| --- | --- | --- | --- | --- | --- | --- | --- | --- | --- | --- | --- |
|  |  | Description child | Affect | | Description of behavior | Affect | | Intrusive | | State/Affekt | |
|  |  |  | + | - |  | + | - | yes | no | + | - |
|  | 1 |  |  |  |  |  |  |  |  |  |  |
|  | 2 |  |  |  |  |  |  |  |  |  |  |
|  | 3 |  |  |  |  |  |  |  |  |  |  |
|  | 4 |  |  |  |  |  |  |  |  |  |  |
|  | 5 |  |  |  |  |  |  |  |  |  |  |
|  | 6 |  |  |  |  |  |  |  |  |  |  |
|  | 7 |  |  |  |  |  |  |  |  |  |  |
|  | 8 |  |  |  |  |  |  |  |  |  |  |
|  | 9 |  |  |  |  |  |  |  |  |  |  |
|  | 10 |  |  |  |  |  |  |  |  |  |  |

**Final rating of the sensitivity scales**

| **Sub-Scales** | **Summary of observation** | **Value** |
| --- | --- | --- |
| **Responsivity**  5 highly responsive – recognizes all signals, also subtle  1 very unresponsive –inattentive, recognizes almost no signal |  |  |
| **Promptness**  5 very promt – almost always f < 3 sec.  1 reactions remain open or very delayed > 3 sec. |  |  |
| **Appropriateness**  5 very appropriate, adequate, child-oriented  1 very inappropriate, not adequate, self-centered |  |  |
| **Intrusiveness**  5 very intrusive – inconsistent, controlling, leading  1 high respect for child autonomy / individuality |  |  |
| **Hostility**  5 much negative affect (anger, hostility, impatience)  1 no negative expression at all |  |  |
| **Positive affect**  5 much positive Affect (smile, laugh, happiness)  1 no positive affect |  |  |
